# Use of Stochastic Simulation to Evaluate the Reduction in Methane Emissions and Improvement in Reproductive Efficiency from Routine Hormonal Interventions in Dairy Herds

**DOI:** 10.1371/journal.pone.0127846

**Published:** 2015-06-10

**Authors:** Simon C. Archer, Christopher D. Hudson, Martin J. Green

**Affiliations:** School of Veterinary Medicine and Science, University of Nottingham, Sutton Bonington, Leicestershire, United Kingdom; University of Alberta, CANADA

## Abstract

This study predicts the magnitude and between herd variation in changes of methane emissions and production efficiency associated with interventions to improve reproductive efficiency in dairy cows. Data for 10,000 herds of 200 cows were simulated. Probability of conception was predicted daily from the start of the study (parturition) for each cow up to day 300 of lactation. Four scenarios of differing first insemination management were simulated for each herd using the same theoretical cows: A baseline scenario based on breeding from observed oestrus only, synchronisation of oestrus for pre-set first insemination using 2 methods, and a regime using prostaglandin treatments followed by first insemination to observed oestrus. Cows that did not conceive to first insemination were re-inseminated following detection of oestrus. For cows that conceived, gestation length was 280 days with cessation of milking 60 days before calving. Those cows not pregnant after 300 days of lactation were culled and replaced by a heifer. Daily milk yield was calculated for 730 days from the start of the study for each cow. Change in mean reproductive and economic outputs were summarised for each herd following the 3 interventions. For each scenario, methane emissions were determined by daily forage dry matter intake, forage quality, and cow replacement risk. Linear regression was used to summarise relationships. In some circumstances improvement in reproductive efficiency using the programmes investigated was associated with reduced cost and methane emissions compared to reliance on detection of oestrus. Efficiency of oestrus detection and the time to commencement of breeding after calving influenced variability in changes in cost and methane emissions. For an average UK herd this was a saving of at least £50 per cow and a 3.6% reduction in methane emissions per L of milk when timing of first insemination was pre-set.

## Introduction

The world population of humans is forecast to exceed 9 billion by 2050 [1]. Most growth is expected in developing countries alongside a trend for increased wealth and urbanisation, leading to unprecedented consumer demand for foods of animal origin [1]. However, livestock are responsible for around 18% of anthropogenic greenhouse gas emissions worldwide [2], and increased agricultural production must be sustainable against a background of climate change and limitations on land availability [3]. Without complete reliance on potentially human-edible food, dairy production can be energetically efficient compared to farming non-ruminants [4]. However microbial ruminal fermentation of cellulosic feeds releases methane, a greenhouse gas with at least 25 times the global warming potential of carbon dioxide [5]. Methane has been identified as an important target for short term climate change mitigation strategies, and thus dairy producers will be required to reduce emissions [6]. However this must occur alongside an increase in productivity and economic efficiency.

Oestrus detection efficacy is a common limiting factor in dairy herd reproductive management, and synchronisation programmes allowing pre-set (fixed time) artificial insemination have become popular, particularly for large herds with limited labour [7]. However routine use of hormonal therapy has been highlighted as an ethical dilemma, and some veterinarians may be reluctant to recommend this approach [8]. As demand for dairy products on the world market increases, milk prices tend to become more volatile [9]. To remain profitable and competitive, farmers may need to both adopt new technologies, and make optimal use of existing ones to reduce the cost of producing milk. Importantly, this should also be achieved with minimal adverse environmental impact.

Using simulations or statistical models to predict herd scenarios is useful to inform decisions around the most appropriate management strategy to adopt [10, 11], particularly where this may be controversial. Existing analyses of hormonal synchronisation programmes in dairy herds have focused on the input of deterministic parameter values, and hence generate the average financial value in a particular case [12–14]. Manual sensitivity analysis is then required to evaluate the impact of changes in uncertain parameters, and this may fail to identify situations that could alter decisions. With stochastic simulation, the importance of variation in input values is explicitly explored [15]. As farming develops in the face of future demands, it is important that common practices can be justified to all interested parties. Despite potentially adverse public perception, hormonal interventions for dairy herds could have benefits to society through reduction in methane emissions in addition to financial benefit for the farmer, and this has not previously been considered. The aim of this study was to predict the magnitude and between herd variation of changes in methane emissions and production efficiency associated with 3 simple hormonal interventions to improve dairy herd reproductive efficiency.

## Materials and Methods

A logistic regression model describing the risk of pregnancy in cows over time after calving was replicated using data from 312 herds (Table 1 [16]). This model was combined with a forward simulation of time series data, as described below, to predict the occurrence of pregnancy following insemination over the course of a cow’s lactation. A 3-level stochastic simulation model was developed using the software R version 3.0.2 [17]. At the highest level, herd data were specified, and used to simulate cow level data. The trajectory of individual cows in each herd was then simulated over 2 years. The model had 4 branches that used the same inputs but varied the approach to reproductive management; a baseline scenario, and 3 potential interventions, such that trajectories for exactly the same simulated cows could be compared under different management programmes.

**Table 1:** Final logistic regression model for the occurrence of pregnancy in cows inseminated at different times.

Fixed effects	Mean coefficient	Standard error
Intercept^1^	-0.517	0.041
Parity = 1	(reference)	
Parity = 2	-0.0136	0.0112
Parity = 3	-0.0209	0.0125
Parity = 4	-0.081	0.014
Parity > = 5	-0.284	0.0122
Days in milk (DIM)	0.0177	0.000875
DIM^^^2	-0.0000994	0.00000616
DIM^3	0.000000177	0.000000013
305 day milk yield^2^	-0.0695	0.00246
Season: October to May	(reference)	
Season: June to September	-0.0861	0.0091
Random effect variance		
Herd level	0.065	0.006
Cow level	0.135	0.005

^1^Parity 1 cow at calving with milk yield of 0 kg.

^2^Estimated cumulative milk yield in 305 days (thousand kg).

### Herd simulation

Measures of production and reproductive efficiency in dairy herds vary, and this could influence the response to management changes. In order to incorporate this variation, independent uniform distributions for herd characteristics were specified where possible based on observed ranges in a dataset of 312 United Kingdom dairy herds (Table 2 [16]). Ten thousand herds were simulated by taking random draws from these distributions to fully explore the parameter space for all joint distributions of herd characteristics.

**Table 2:** Herd level inputs for simulation model.

Parameter	Distribution
Submission risk^1^	Uniform(0.1, 0.65)
Pregnancy risk^2^	Uniform(0.1, 0.65)
Milk yield^3^	Uniform(3, 10)
Heifers^4^	Uniform (0.05, 0.5)
Calving index cost (£/day)^5^	Uniform (2, 5)
Cull cost^6^	Uniform(700, 1500)
Serve cost^7^	Uniform(0, 25)
VWP^8^	Uniform(30, 70)
Time to first cycle (days)	Uniform(20, 30)
Cycle length^9^	Uniform(20.5, 22.5)
Forage ME^10^	Uniform(9.5, 11.5)
Milk margin (£/L)	Uniform(0.1, 0.2)

^1^Proportion of eligible cows that are served prior to intervention.

^2^Proportion of served cows that conceive prior to intervention.

^3^Cumulative 305 day milk yield (thousand kg).

^4^Proportion of heifers in the herd.

^5^Cost of changing the interval between subsequent calvings.

^6^Depreciation cost of culling a cow and replacing with a heifer (£).

^7^Cost of each artificial insemination (£).

^8^Voluntary waiting period (days) from calving to first service.

^9^Length of oestrous cycle (days).

^10^Metabolisable energy (MJ/kg dry matter).

### Cow simulation

Distributions of cow characteristics depend on the characteristics of the herd they are in. Therefore, data for 200 cows in each herd were generated by taking random draws from the distributions in Table 2. The parity of cows varied according to the observed age structure in a previous dataset, and this influenced cumulative milk yield over a standardised 305 day lactation [16]. Beta distributions were used to simulate physiological variation in length of post-partum anoestrus (mode = herd average, minimum = 10, maximum = 80), and oestrous cycle length in days (mode = herd average, minimum = 18, maximum = 27 [18]).

### Lactation simulation

Cows cycle between productive periods of lactation and non-productive (dry) periods in late pregnancy, dependent on the timing of re-breeding. In this study individual cows were followed up over 730 days commencing with the birth of a calf (calving) and a period of lactation. Repeated days of lactation for each cow were represented as lines in an array, the initial calving was equally likely to occur on any day of the year. At the start of lactation, cows’ ovaries are inactive for a variable time before cycles commence (post-partum anoestrus). Oestrus refers to a sexually receptive stage of the ovarian cycle characterised by physiological and behavioural changes [19]. Therefore day of the ovarian cycle was determined based on a period of post-partum anoestrus followed by the recurrence of regular length cycles (both based on random draws from input distributions). Cows were deemed to be in oestrus on day 1 of the cycle, and an insemination event was simulated at random according to the distribution of risk that the cow was observed in oestrus and inseminated (submission risk), provided that the designated voluntary post parturient non-breeding period (voluntary waiting period) for the herd had elapsed. The outcome of insemination (pregnant or not pregnant) was determined by a random draw from a distribution based on the mean predicted probability of conception on that day (Table 1). Breeding continued until the occurrence of pregnancy or day 300 of lactation. Daily milk yield was calculated based on stage of lactation, time of year and pregnancy status using a previously reported method [20]. Pregnant cows were deemed to have a physiologically normal gestation length of 280 days and milking ceased 60 days before their expected calving date. Cows not pregnant by day 300 were culled and replaced by a young cow (heifer) after a lag time of 60 days.

Methane emissions arise from the ruminal fermentation of cellulose which is relatively more important in late lactation when forage feeds (such as grass silage) make up a higher proportion of the cows’ diet. It was assumed that forage alone provided energy for maintenance and up to 10 L of milk production per day. To support higher milk yields, concentrate was assumed to be fed at 0.4 kg per L and this was 90 per cent dry matter. Daily dry matter intake was calculated from the stage of lactation and milk yield as reported previously [21]. Methane emissions were mainly determined by daily forage dry matter intake (total dry matter intake minus concentrate dry matter intake), forage quality, and replacement risk [22]. The proportion of herd methane emissions produced by replacement heifers was estimated to be 27% under commercial conditions [22]. Methane production from concentrate feeds occurred, but this was 150 times lower than for forage feeds on a dry matter basis [22].

This baseline scenario (Group 1) was compared directly to simulations that incorporated one of three hormonal interventions that altered the timing of first insemination, submission and pregnancy risk. These calculations were made in parallel with the baseline scenario using the same theoretical cows and are described below.

### Group 2: Ovsynch

The Ovsynch protocol involves 2 injections with gonadotrophin releasing hormone analogues given 9 days apart, and a prostaglandin injection on day 7. This protocol is used to ensure all cows receive a first insemination at a fixed time (16 to 24 hours after the completion of the protocol), which eases and automates reproductive management of dairy herds [7, 23, 24]. The programme of injections was assumed to be applied in order to schedule a fixed time insemination from day 50 of lactation (50 DIM). In practice, it is preferable to administer routine treatments to groups of eligible cows rather than individuals. It was therefore assumed that treatments took place every 2 weeks, and cows were therefore inseminated between 50 and 64 DIM, and day of the oestrus cycle was set to 1 on the treatment day. This change was made regardless of the herd voluntary waiting period, and all cows were served (submission risk = 1). Probability of becoming pregnant at insemination (conception risk) was altered by a factor drawn from a beta distribution (mode = 0.8, minimum = 0.4, maximum = 1.7) obtained from a previous study [25]. Cows that did not become pregnant to first insemination continued regular oestrous cycles and repeated inseminations as with the baseline scenario until the occurrence of pregnancy or 300 DIM. Cows that were still in the postpartum anoestrus period by 64 DIM, having not resumed regular cycles were assumed to be infertile and could not conceive despite treatment and insemination.

### Group 3: Ovsynch with progesterone

This regime was similar to the Ovsynch protocol above but progesterone was also administered to all cows, via a controlled intravaginal drug releasing device for a 7 day period commencing at the start of the protocol. This therapy was investigated as it has been associated with beneficial influences on subsequent fertility in anoestrus cows compared to Ovsynch alone [26–30]. Specifically, this was summarised as a 5% increase in the risk of resumption of cyclicity by 64 DIM for multiparous cows that had not commenced regular cyclicity in the baseline scenario, based on reported research with metrics suitable for inclusion [29]. First insemination submission risk was 1 (as for Ovsynch), and the baseline pregnancy risk was altered by a factor drawn from a beta distribution (mode = 0.96, minimum = 0.96, maximum = 1.02) obtained from a previous study [30]. Cows that did not become pregnant to first insemination continued regular oestrous cycles and repeat inseminations as for the baseline scenario until the occurrence of pregnancy or 300 DIM.

### Group 4: Double prostaglandin

In this scenario, hormonal (prostaglandin) injections were only administered to cows that had not already been inseminated by a specified time after calving. Eligible cows were treated in batches every 2 weeks such that oestrus could occur from 50 DIM, assuming regular ovarian cycles had resumed. The probability that prostaglandin treatment resulted in oestrus was taken to be 0.8 [31]. Following successful treatments, cows were simulated as being observed in oestrus according to the herd submission risk and were then simulated as being inseminated. Otherwise a second injection was administered 2 weeks later, and the process was repeated. Pregnancy risk given prostaglandin treatment, oestrus observation, and insemination was altered by a factor drawn from a uniform distribution (minimum = 0.9, maximum = 1.1) based on summary results from 2 contradictory studies [31, 32]. Cows that did not become pregnant, or which underwent the double prostaglandin regime without being inseminated were then handled as described for the baseline situation.

### Herd summary

The absolute differences between mean parameter values were determined by subtracting the mean estimate calculated for each intervention scenario from the mean estimate calculated for the baseline scenario. This was used to determine the mean change in non-tangible costs based on the mean change in milk yield, mean change in the number of cows served, and differences in the proportions of cows culled, multiplied by the respective unit costs (Table 2). The change in costs modelled did not include the costs of implementing the programme as drug costs would be known by the farmer. For comparison, drug costs per cow were taken as: Ovsynch; £9, Ovsynch with progesterone; £19, double prostaglandin; £5. The proportion of cows in each herd predicted to have not resumed regular oestrous cycles by the end of the herd voluntary waiting period was recorded.

### Associations between inputs and outputs

Scatter plots were used to visualise relationships between input parameters and the change in cost (based on changes in milk production, culling, and insemination costs) or methane emissions with each intervention. Multivariate analyses were then applied; 6 linear regression models were developed (2 for each intervention) with difference in cost /cow per year or herd methane emissions (g per L milk produced) as outcomes and herd as random effects. Models were built in MLwiN version 2.29 [33]. Mean coefficient values were generated with the iterative generalised least squares algorithm. All parameters in Table 2 were investigated for inclusion in the model as polynomials. Parameters were removed from a saturated model if their mean effect size was ≤ the standard error (Wald test; *P* ≤ 0.05). Biologically plausible interactions between remaining parameters were investigated. Model fit assessment was by inspection of the residuals [33]

## Results

### Multivariate analyses of change in methane emissions

**Fig 1:**
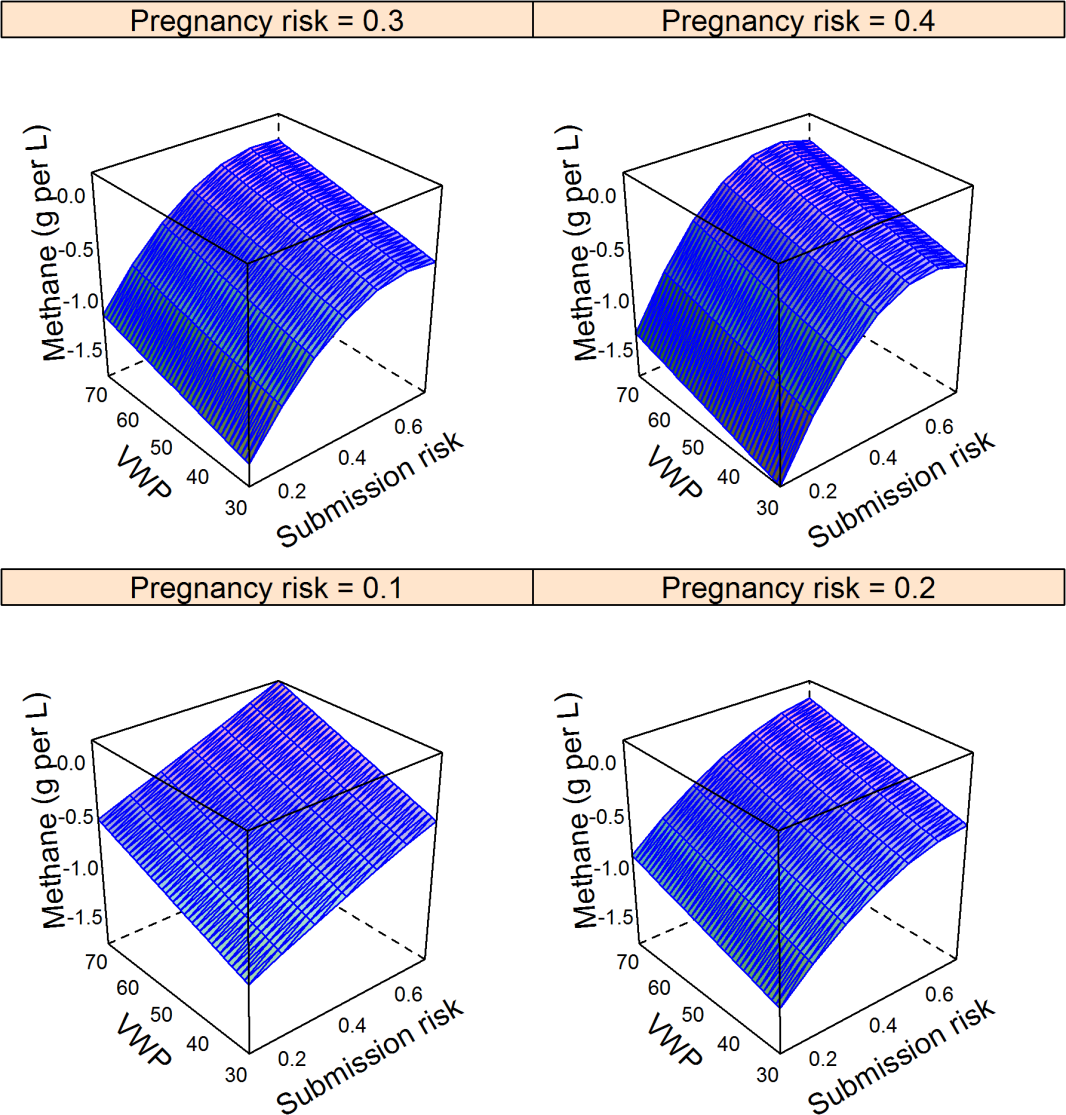
Model predictions of mean change in methane emissions (g per L of milk produced) through the Ovsynch programme in herds with varying voluntary post-partum non-breeding time (VWP), risk for cows being identified in oestrus and inseminated, and pregnancy risk compared to reliance on oestrus observations. Data for 10,000 herds of 200 cows were simulated (Table 2). Methane emissions per cow were estimated from daily forage dry matter intake, forage quality, and replacement risk. Cumulative milk yield per cow was estimated based on parity, stage of lactation, and stage of gestation. Methane emissions in the baseline scenario were subtracted to give the expected change in methane per L milk produced. Negative values indicate reductions. Associations with input parameters were evaluated in a linear model (Table 3); mean values were used to generate predictions.

**Fig 2:**
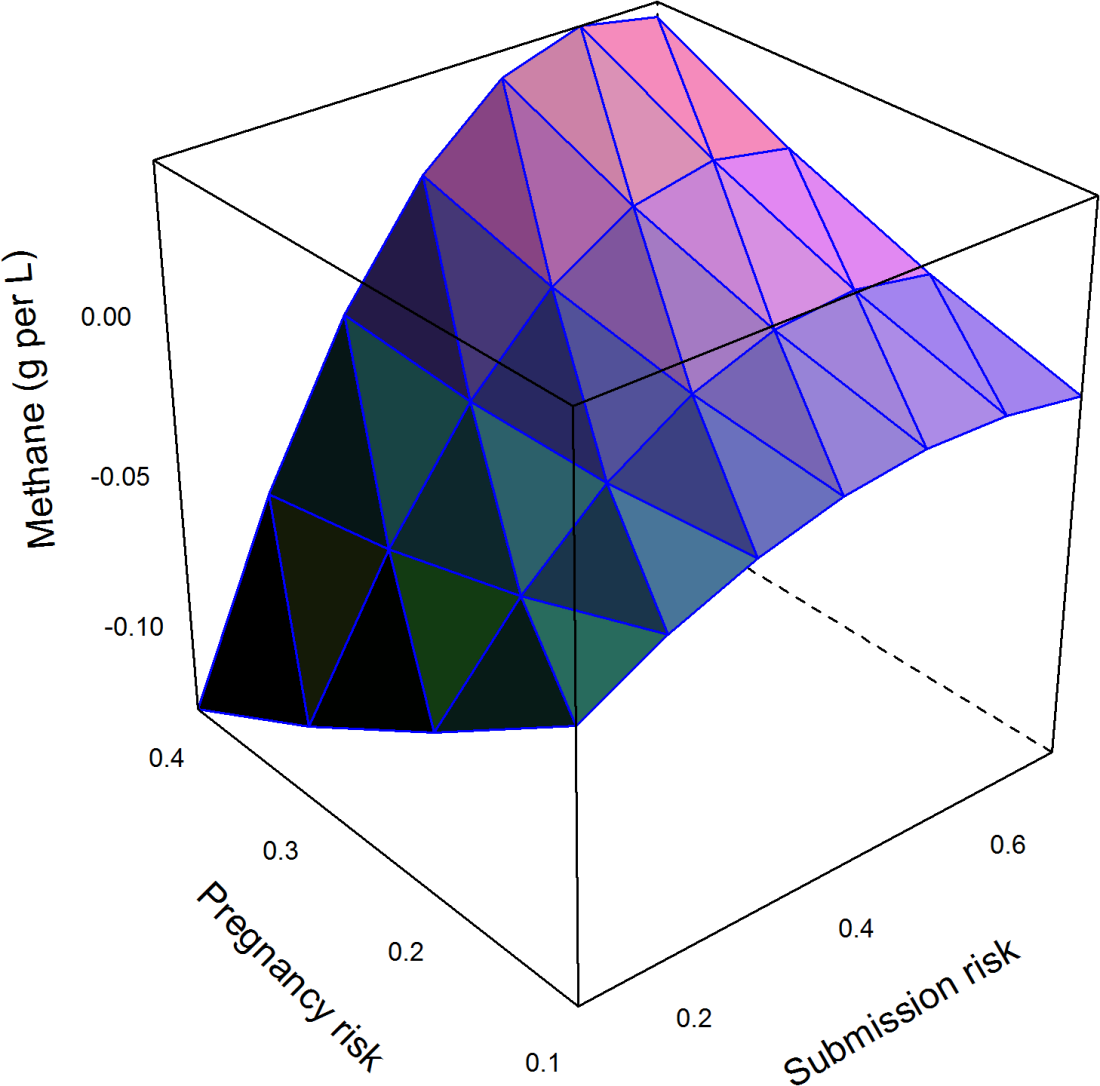
Model predictions of mean marginal change in methane emissions (g per L of milk produced) through supplementing the Ovsynch programme with progesterone in herds with varying, risk for cows being identified in oestrus and inseminated, and pregnancy risk following insemination. Data for 10,000 herds of 200 cows were simulated (Table 2). Methane emissions per cow were estimated from daily forage dry matter intake, forage quality, and replacement risk. Cumulative milk yield per cow was estimated based on parity, stage of lactation, and stage of gestation. Associations with input parameters were evaluated in a linear model (Table 3). Mean predicted methane emissions in the Ovsynch scenario were subtracted from that for use of Ovsynch with supplementary progesterone to give the expected change in methane per L milk produced. Negative values indicate reductions.

**Fig 3:**
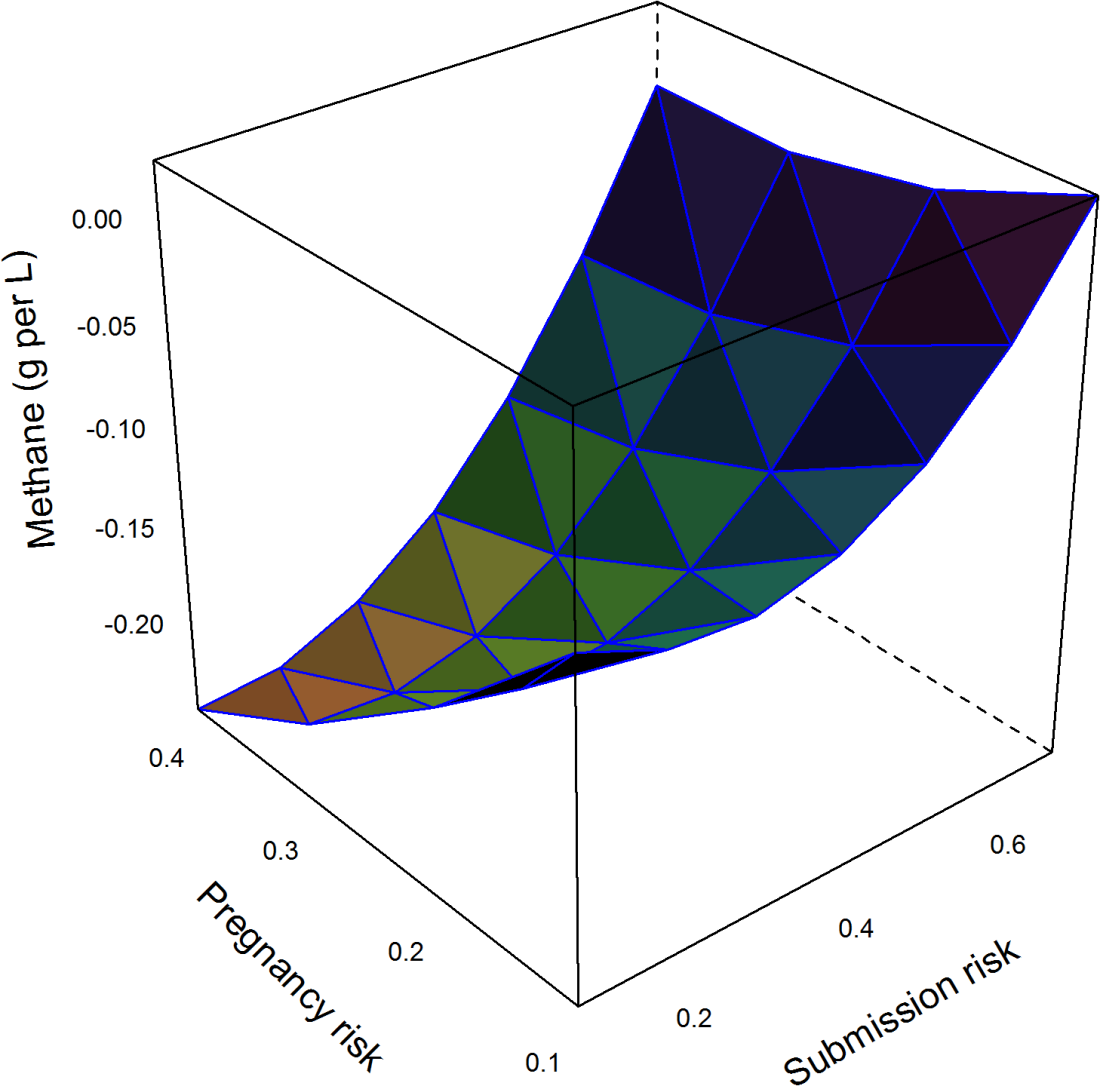
Model predictions of mean change in methane emissions (g per L of milk produced) through treatment of cows with up to 2 prostaglandin injections separated by an interval of 2 weeks prior to first insemination by 64 days in milk in herds with varying risk for cows being identified in oestrus and inseminated, and pregnancy risk compared to reliance on oestrus detection. Data for 10,000 herds of 200 cows were simulated (Table 2). Methane emissions per cow were estimated from daily forage dry matter intake, forage quality, and replacement risk. Cumulative milk yield per cow was estimated based on parity, stage of lactation, and stage of gestation. Methane emissions in the baseline scenario were subtracted to give the expected change in methane per L milk produced. Negative values indicate reductions. Associations with input parameters were evaluated in a linear model (Table 3); mean values were used to generate predictions.

The major factors that determined changes in methane emissions are shown in the final models in Table 3. These models explained 36%, 41%, and 4% of the null model variance for the Ovsynch, Ovsynch with progesterone, and double prostaglandin programmes respectively; residuals were distributed normally (Table 3). The impact of comparable changes in model input parameters (from the mean to the upper quartile) on change in methane emissions per L of milk with other inputs held at the mean is shown in Table 4; the Ovsynch based programmes (Groups 2 and 3) were most beneficial in herds with low submission risk, pregnancy risk, and voluntary waiting period. However the models included quadratic terms and interactions (Table 3) meaning the largest reductions in methane emissions per L of milk produced occurred in herds with the lowest submission risks, but otherwise good reproductive efficiency (voluntary waiting period < 50 days and high pregnancy risk; Fig 1). Further reductions in methane occurred through supplementing the Ovsynch programme (Group 2) with progesterone (Group 3) that increased with increasing pregnancy risk and decreasing submission risk (Fig 2). A similar trend was observed for the reduction in methane emissions associated with the double prostaglandin treatment (Fig 3), although the absolute reduction in methane was less than for the other programmes.

**Table 3:** Final models for the difference in methane emissions (g) per litre of milk produced following the implementation of 3 reproductive management programmes in dairy herds compared to a baseline^1^ scenario.

	Group 2: Ovsynch^2^		Group 3: Ovsynch with progesterone^3^		Group 4: Double prostaglandin^4^	
**Fixed effects**	Mean	SE^5^	Mean	SE	Mean	SE
Intercept	-0.5	0.0	-0.5	0.0	-0.2	0.0
(Submission risk)^6^	2.4	0.0	2.7	0.0	0.3	0.0
(Submission risk).(Submission risk)	-5.2	0.3	-5.8	0.3	0.7	0.2
(Pregnancy risk)^7^	0.5	0.1	0.6	0.1	-0.2	0.0
(Pregnancy risk).(Pregnancy risk)	2.0	0.3	2.2	0.3	0.8	0.2
(Milk yield)^8^	0.0	0.0	0.0	0.0		
(VWP)^9^	0.0	0.0	0.0	0.0	0.0	0.0
(Submission risk).(VWP)	0.0	0.0	0.0	0.0		
(Submission risk).(Submission risk).(VWP)	0.0	0.0	0.1	0.0		
(Pregnancy risk).(VWP)	0.0	0.0	0.0	0.0		
(Pregnancy risk).(Pregnancy risk).(VWP)	0.1	0.0	0.1	0.0		
(Pregnancy risk).(Submission risk)	4.59	0.2	5.5	0.2	0.7	0.2
(Pregnancy risk).(Submission risk)^2	-19.5	1.6	-21.1	1.6		
**Random effects**	Variance	SE	Variance	SE	Variance	SE
Herd	0.3	0.0	0.3	0.0	0.2	0.0

^1^ Cows submitted for service based on observation of oestrus only (Group 1).

^2^ All cows treated with gonadotropin releasing hormone and prostaglandin such that they all receive a first service between 50 and 64 days in milk regardless of oestrous detection. Repeat services based on observation of oestrous.

^3^ All cows treated with gonadotrophin releasing hormone, progesterone, and prostaglandin such that all cows can receive a first service between 50 and 64 days in milk. Repeat services based on observation of oestrus.

^4^ Cows not observed in oestrous treated up to 2 times with prostaglandin to increase the probability of oestrus between 50 and 64 days in milk. All services based on observation of oestrus.

^5^ Standard error.

^6^ Proportion of eligible cows that are served prior to intervention (centred on the mean). Polynomial term.

^7^ Proportion of served cows that conceive prior to intervention (centred on the mean). Polynomial term.

^8^ Cumulative 305 day milk yield (thousand kg; centred on the mean).

^9^ Voluntary waiting period (days) from calving to first service (centred on the mean).

**Table 4:** Model predictions; impact of a change in input values from the mean to the upper quartile at mean values of other parameters, on the difference in methane emissions (g /litre of milk produced) following the implementation of 3 reproductive management programmes in dairy herds compared to a baseline^1^ scenario; negative values indicate reductions.

	Size of change (mean to upper quartile)	Group 2: Ovsynch^2^	Group 3: Ovsynch with progesterone^3^	Group 4: Double prostaglandin^4^
(Submission risk)^5^	0.37 to 0.51	0.23	0.23	0.06
(Pregnancy risk)^6^	0.37 to 0.51	0.11	0.13	-0.01
(Milk yield)^7^	6.5 to 8.3	0.01	0.01	
(VWP)^8^	50 to 60	0.06	0.06	0.03

^1^Cows submitted for service based on observation of oestrus only (Group 1).

^2^ All cows treated with gonadotropin releasing hormone and prostaglandin such that they all receive a first service between 50 and 64 days in milk regardless of oestrous detection. Repeat services based on observation of oestrus.

^3^ All cows treated with gonadotrophin releasing hormone, progesterone, and prostaglandin such that all cows can receive a first service between 50 and 64 days in milk. Repeat services based on observation of oestrus.

^4^ Cows not observed in oestrus treated up to 2 times with prostaglandin to increase the probability of a heat between 50 and 64 days in milk. All services based on observation of oestrus.

^5^ Proportion of eligible cows that are served prior to intervention (centred on the mean). Polynomial term.

^6^ Proportion of served cows that conceive prior to intervention (centred on the mean). Polynomial term.

^7^ Cumulative 305 day milk yield (thousand kg; centred on the mean).

^8^ Voluntary waiting period (days) from calving to first service (centred on the mean).

### Multivariate analyses of change in costs

**Fig 4:**
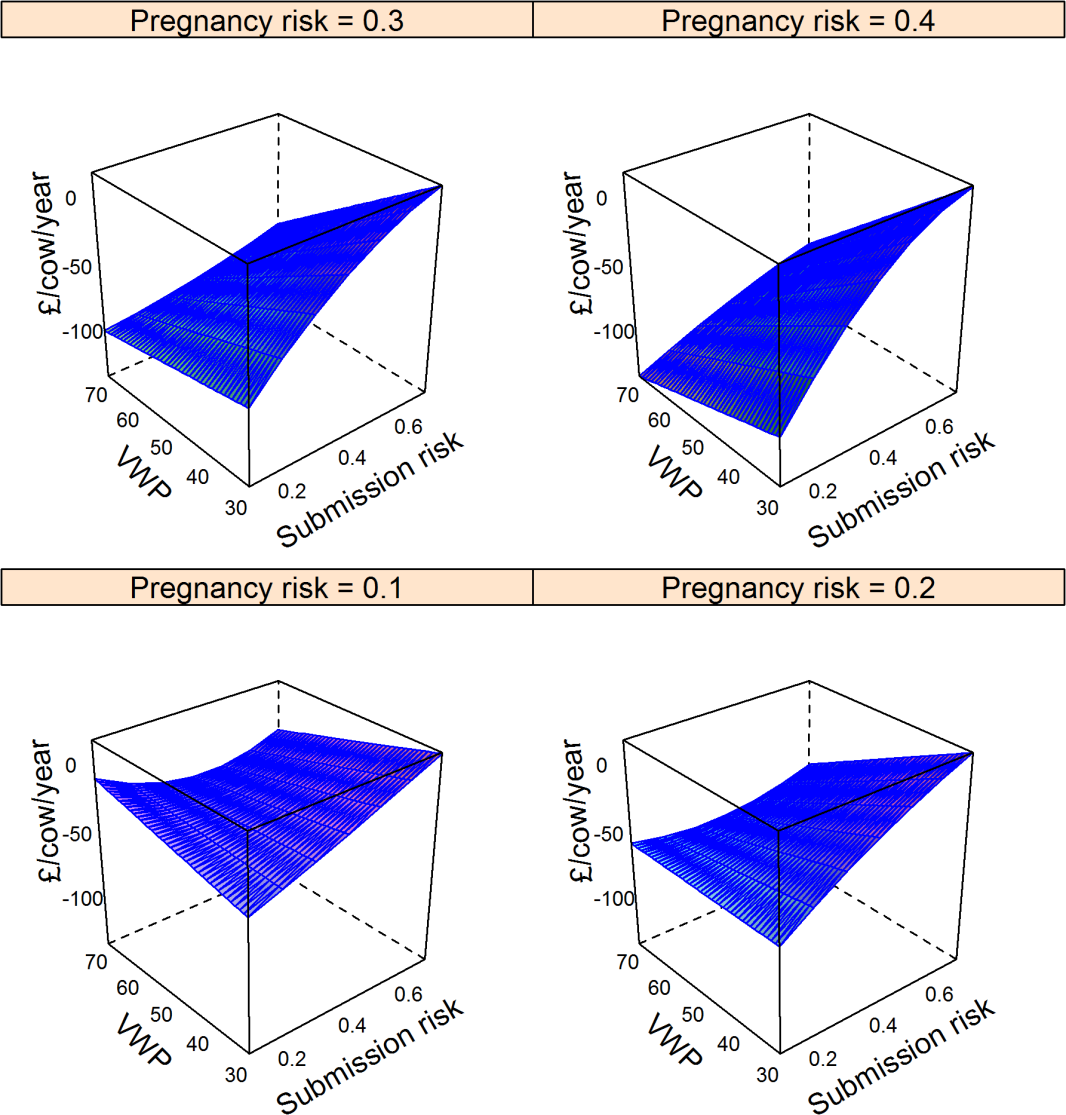
Model predictions of mean change in cost (£/cow/year) through the Ovsynch programme in herds with varying voluntary post-partum non-breeding time (VWP), risk for cows being identified in oestrus and inseminated, and pregnancy risk compared to reliance on oestrus detection. Data for 10,000 herds of 200 cows were simulated (Table 2). The cost /cow per year was determined from the proportion of cows that were culled due to failure to conceive by 300 days in milk, the number of inseminations required, and the difference in cumulative milk yield per cow. Cost of the baseline scenario was subtracted to give the expected change in costs. Negative values indicate financial gain. Associations with input parameters were evaluated in a linear model (Table 5); mean values were used to generate predictions.

**Fig 5:**
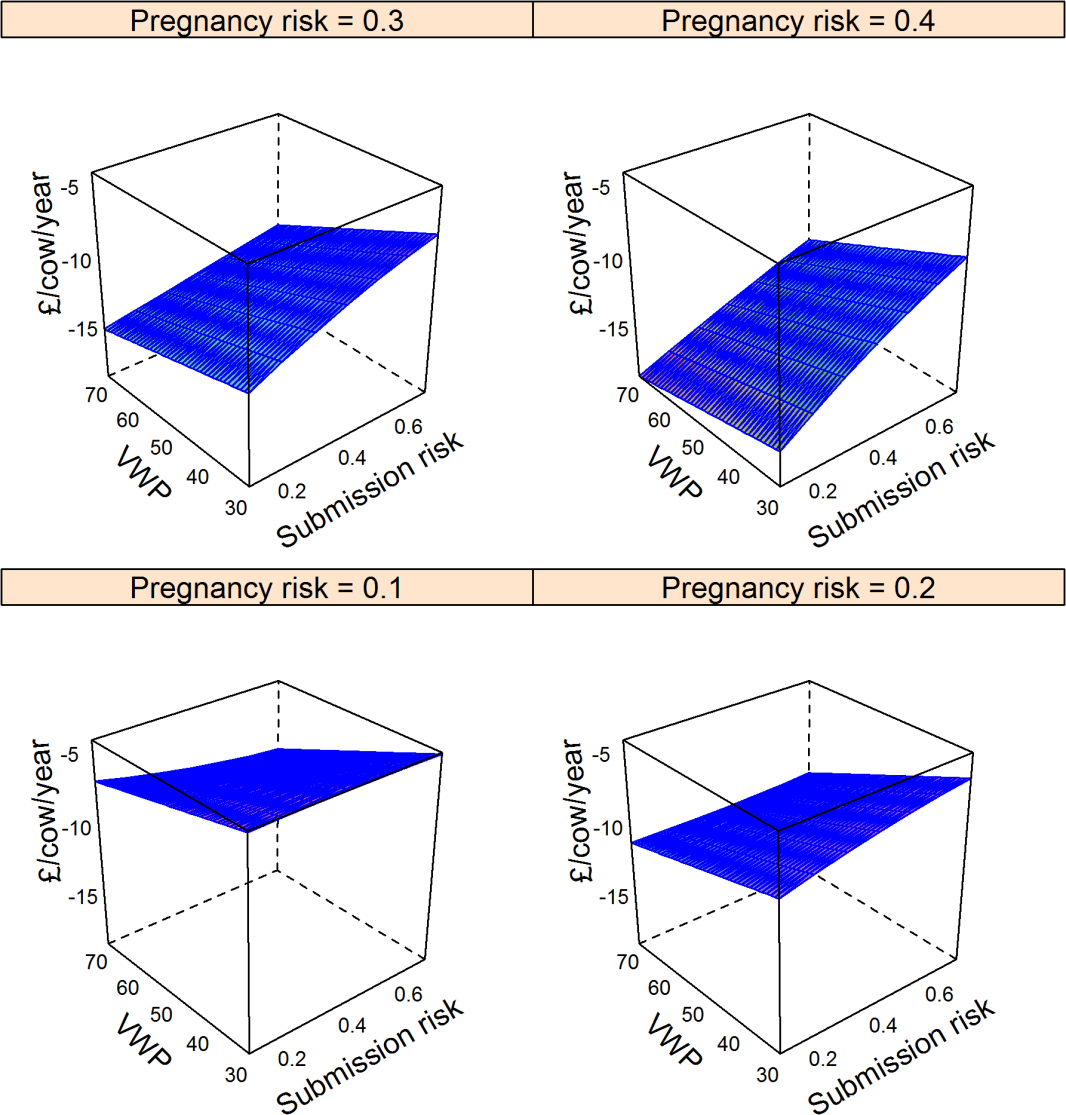
Model predictions of mean marginal change in cost (£/cow/year) through supplementing the Ovsynch programme with progesterone in herds with varying voluntary post-partum non-breeding time (VWP), risk for cows being identified in oestrus and inseminated, and pregnancy risk following insemination. Data for 10,000 herds of 200 cows were simulated (Table 2). The cost of each programme /cow per year was determined from the proportion of cows that were culled due to failure to conceive by 300 days in milk, the number of inseminations required, and the difference in cumulative milk yield per cow. Associations with input parameters were evaluated in a linear model (Table 5). The mean predicted cost of the Ovsynch scenario was subtracted from that for use of Ovsynch with supplementary progesterone to give the expected marginal change in costs. Negative values indicate financial gain.

**Fig 6:**
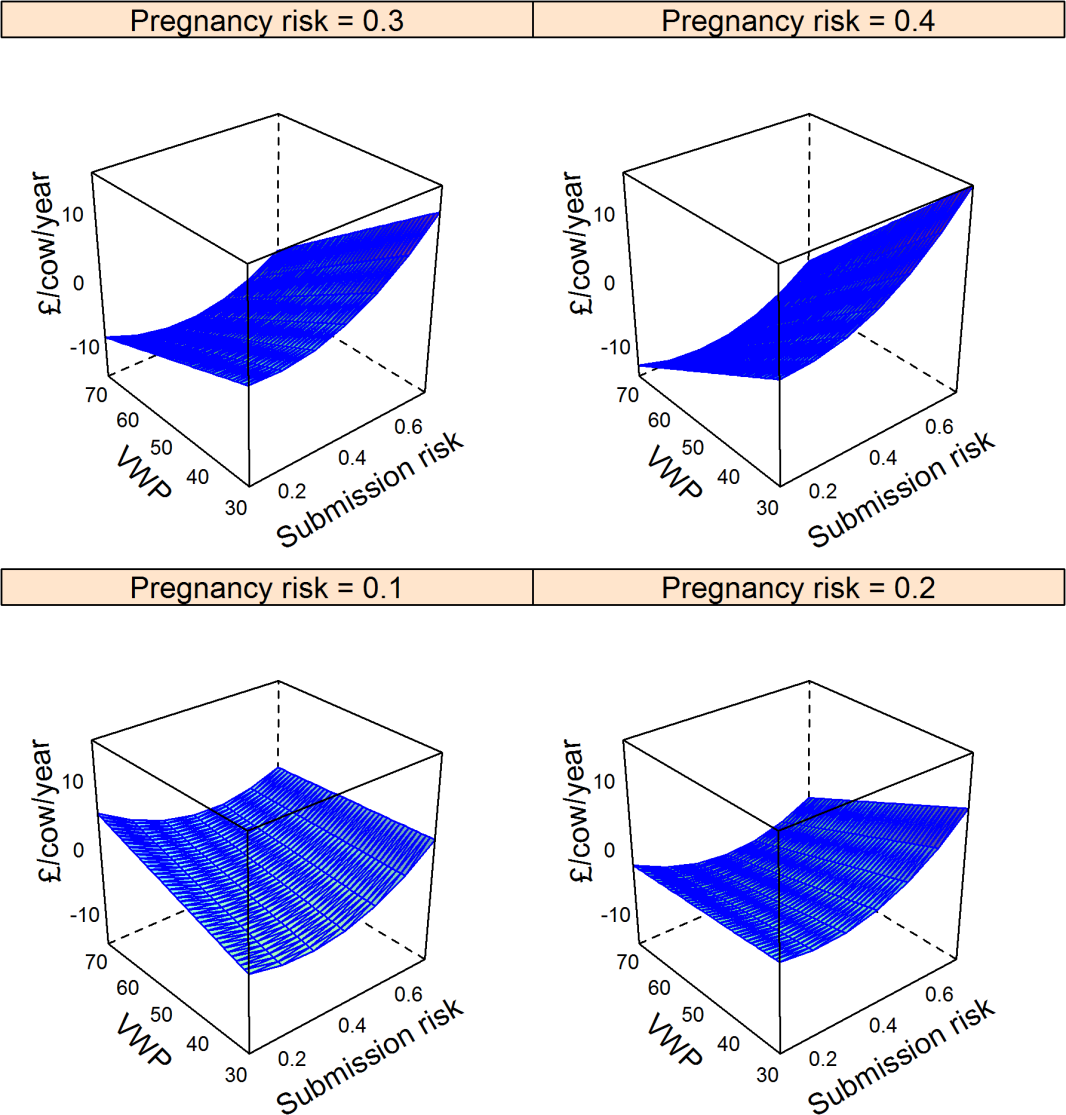
Model predictions of mean change in cost (£/cow/year) through treatment of cows with up to 2 prostaglandin injections separated by an interval of 2 weeks prior to first insemination by 64 days in milk in herds with varying voluntary post-partum non-breeding time (VWP), risk for cows being identified in oestrus and inseminated compared to reliance on oestrus observations. Data for 10,000 herds of 200 cows were simulated (Table 2). The cost /cow per year was determined in each scenario from the proportion of cows that were culled due to failure to conceive by 300 days in milk, the number of inseminations required, and the difference in cumulative milk yield per cow. Cost of the baseline scenario was subtracted to give the expected change in costs. Negative values indicate financial gain. Associations with input parameters were evaluated in a linear model (Table 5); mean values were used to generate predictions.

The major factors that determined changes in cost are shown in the final models in Table 5. These models explained 65%, 68%, and 8% of the null model variance for the Ovsynch, Ovsynch with progesterone, and double prostaglandin programmes respectively; residuals were distributed normally (Table 5). The impact of comparable changes in model input parameters (from the mean to the upper quartile) on change in costs with other inputs held at the mean is shown in Table 6. The hormonal interventions were all most financially beneficial if herd voluntary waiting period exceeded 50 days. For the Ovsynch based programmes (Groups 2 and 3; Table 6), comparable changes in submission and pregnancy risk, at mean values of other inputs were associated with similar magnitude of change in cost but in opposing directions, indicating that these approaches would be economically beneficial in herds with low submission risk, but relatively high pregnancy risk. Ovsynch programmes were more economically beneficial when the depreciation cost of cull cows increased (Table 6). Cost savings through the Ovsynch programme (Group 2) exceeded drug costs except if submission risk exceeded 0.5 (Fig 4). Results for supplementing the Ovsynch programme with progesterone (Group 3) were similar to use of Ovsynch alone (Group 2); therefore the marginal benefit of progesterone supplementation is shown in Fig 5. With other inputs held at the mean, cost savings through progesterone supplementation failed to exceed the marginal cost of treatment for herds with pregnancy risk < 0.2, or submission risks > 0.5, otherwise the decision would depend on the balance of voluntary waiting period and submission risk (Fig 5). Comparable multivariate plots for the double prostaglandin programme confirm that cost savings may fail to exceed drug costs unless the herd voluntary waiting period is reduced (Fig 6).

**Table 5:** Final models for the difference in cost per cow (£ /year; based on change in milk production, culling risk, and number of services) following the implementation of 3 reproductive management programmes in dairy herds compared to a baseline^1^ scenario.

	Group 2: Ovsynch^2^		Group 3: Ovsynch with progesterone^3^		Group 4: Double prostaglandin^4^	
**Fixed effects**	Mean	SE^5^	Mean	SE	Mean	SE
Intercept	-64.7	0.6	-78.0	0.6	-5.5	0.4
(Submission risk)^6^	134.3	2.0	142.8	2.0	11.6	1.3
(Submission risk).(Submission risk)	-103.3	13.8	-106.3	14.1	44.5	9.0
(Pregnancy risk)^7^	-128.7	3.0	-155.5	3.0	-5.9	1.3
(Pregnancy risk).(Pregnancy risk)	112.3	13.9	120.0	14.2	29.2	9.1
(Milk yield)^8^	-1.2	0.2	-1.4	0.2		
(VWP)^9^	-1.9	0.1	-1.9	0.1	0.0	0.0
(Cull cost)^10^	-0.1	0.0	-0.1	0.0		
(Serve cost)^11^	0.4	0.0	0.3	0.0		
(Submission risk).(VWP)	-3.1	0.2	-3.2	0.2	-0.4	0.1
(Submission risk).(Submission risk).(VWP)	4.6	1.2	4.7	1.2		
(Pregnancy risk).(VWP)	-3.9	0.2	-3.8	0.2	-1.3	0.1
(Pregnancy risk).(Pregnancy risk).(VWP)	7.2	1.2	7.6	1.2	2.5	0.8
(Pregnancy risk).(Submission risk)	404.4	12.4	446.0	12.4	46.1	8.0
(Pregnancy risk).(Submission risk)^2	-810.3	88.5	-825.2	88.5		
**Random effects**	Variance	SE	Variance	SE	Variance	SE
Herd	976.8	13.8	1023.3	14.5	420.9	6.0

^1^ Cows submitted for service based on observation of oestrus only (Group 1).

^2^ All cows treated with gonadotropin releasing hormone and prostaglandin such that they all receive a first service between 50 and 64 days in milk regardless of oestrous detection. Repeat services based on observation of oestrus.

^3^ All cows treated with gonadotrophin releasing hormone, progesterone, and prostaglandin such that all cows can receive a first service between 50 and 64 days in milk. Repeat services based on observation of oestrus.

^4^ Cows not observed in oestrous treated up to 2 times with prostaglandin to increase the probability of a heat between 50 and 64 days in milk. All services based on observation of oestrus.

^5^ Standard error.

^6^ Proportion of eligible cows that are served prior to intervention (centred on the mean). Polynomial term.

^7^ Proportion of served cows that conceive prior to intervention (centred on the mean). Polynomial term.

^8^ Cumulative 305 day milk yield (thousand kg; centred on the mean).

^9^ Voluntary waiting period (days) from calving to first service (centred on the mean).

^10^ Cost of culling a cow and replacing with a heifer (£; centred on the mean).

^11^ Cost of each artificial insemination (£; centred on the mean).

**Table 6:** Model predictions; impact of a change in input values from the mean to the upper quartile at mean values of other parameters, on the difference in cost per cow (£ /year; based on change in milk production, culling risk, and number of services) following the implementation of 3 reproductive management programmes in dairy herds compared to a baseline^1^ scenario; negative values indicate financial gain.

	Size of change (mean to upper quartile)	Group 2: Ovsynch^2^	Group 3: Ovsynch with progesterone^3^	Group 4: Double prostaglandin^4^
(Submission risk)^5^	0.37 to 0.51	16.2	17.3	2.4
(Pregnancy risk)^6^	0.37 to 0.51	-15.3	-18.8	-0.3
(Milk yield)^7^	6.5 to 8.3	-2.1	-2.4	
(VWP)^8^	50 to 60	-18.8	-19.4	-4.4
(Cull cost)^9^	1100 to 1300	-12.3	-14.2	-1.8
(Serve cost)^10^	12 to 19	2.5	2.1	

^1^ Cows submitted for service based on observation of oestrus only (Group 1).

^2^ All cows treated with gonadotropin releasing hormone and prostaglandin such that they all receive a first service between 50 and 64 days in milk regardless of oestrus detection. Repeat services based on observation of oestrus.

^3^ All cows treated with gonadotrophin releasing hormone, progesterone, and prostaglandin such that all cows can receive a first service between 50 and 64 days in milk. Repeat services based on observation of oestrus.

^4^ Cows not observed in oestrous treated up to 2 times with prostaglandin to increase the probability of a heat between 50 and 64 days in milk. All services based on observation of oestrus.

^5^ Proportion of eligible cows that are served prior to intervention (centred on the mean). Polynomial term.

^6^ Proportion of served cows that conceive prior to intervention (centred on the mean). Polynomial term.

^7^ Cumulative 305 day milk yield (thousand kg; centred on the mean).

^8^ Voluntary waiting period (days) from calving to first service (centred on the mean).

^9^ Cost of culling a cow and replacing with a heifer (£; centred on the mean).

^10^ Cost of each artificial insemination (£; centred on the mean).

## Discussion

Across a wide range of herd scenarios, use of hormonal therapy to aid reproductive management of dairy cows can lead to economic and environmental benefits. However the scale of variability between herds in these outcomes emphasises that decisions around changing reproductive management require careful consideration. Where controversy over interventions exists it is important that use is justified to be in the public interest, in addition to being economically beneficial to the farmer. From this study, society could benefit through judicious application of hormonal programmes to specific herds through availability of affordable milk, and a relative reduction in methane emissions. These issues are increasingly important due to expected population growth, and the challenge for the dairy industry will be to engage positively with consumers to maintain support for development. Average UK herd size and annual milk yield per cow are 126 and 7,353 L respectively (with increasing trends [34]). If such a herd also had average input values (Table 2), the 3.6% annual reduction in methane (0.4 g per L of milk) if an Ovsynch programme was used prior to first insemination compared with breeding to observed oestrus, would be roughly equivalent to the annual global warming potential of 2 cars, a family home, or 21 barrels of oil [5, 35]. The synchronisation programmes tested could be put in place quickly, with benefits after 1 year. This is consistent with targeting of methane for short term greenhouse gas emission reductions that may be attractive for policy makers [6]. Changing farm management depends on the compliance of the farmer which could be facilitated by an understanding of the costs involved; a gain of £50 per cow after deduction of drug costs for the average case described. This saving could ultimately also benefit society if milk is more available or becomes cheaper as a result. However, it is not clear how to quantify the opinion of society on use of routine hormone therapy to balance against change in methane emissions and costs. This problem has also occurred in human medicine, where quality adjusted life years are used as units in cost effectiveness analyses; however an arbitrary monetary value must still be assigned to these units for comparison with treatment costs [36].

The synchronisation programmes tested were not always associated with clear benefits for all interested parties. This emphasises the benefit of using stochastic simulation for predictions to investigate all feasible scenarios. For example, if a herd with upper quartile submission risk, pregnancy risk and voluntary waiting period, but otherwise average performance applied an Ovsynch programme, there would be no decrease in methane emissions per L of milk produced (Fig 1), yet the farmer would be better off (Fig 4). In this situation, routine hormone therapy may not be accepted by society. Conversely, an Ovsynch programme could reduce methane emissions per L of milk for a herd with high submission risk and a short voluntary waiting period despite no direct financial benefit to the farmer (Fig 1; Fig 4). This could occur if reproductive efficiency improved to such an extent that the number of cows that were dry per day of the study increased, meaning less milk was sold. Prompt breeding and short lactations imply that forage intake and hence methane emissions per L of milk would decline.

We assumed that inputs and outputs for individual herds were known. With the exception of the double prostaglandin programme that was most similar to the baseline scenario, linear models explained most variation between herds; the remainder was due to the complex manner in which uncertainty in parameters was propagated through the simulation models [15]. However in reality there is likely to be both epistemic uncertainty in input values (which can also vary over time), and aleatory uncertainty in outcomes that have yet to occur at the point of making the decision. In practice, it is therefore important to make calculations for specific farms rather than rely on generalisations to inform decision making around management programmes using hormonal therapy. These should also be tailored to specific situations. From the farmers’ point of view, it is simplistic to assume that it is acceptable to at least break even, and some decision makers may expect a higher level of return to compete with other investments. The impact of the attitude to risk and willingness to pay of decision makers has been used to set budgets for interventions to control mastitis [10], but not to aid decisions around reproductive management, which are potentially of greater economic importance. It is therefore useful to present the likely impact of interventions and demonstrate the potential scale of effects to producers, and indicate how likely it is that these will occur. This is particularly important for marginal benefits such as for supplementing the Ovsynch programme with progesterone. A similar approach could be applied to reduction in methane emissions to justify use of hormone treatments to society, although it is not clear what level of reduction would be sufficient.

Despite simulation of a positive influence of Ovsynch with progesterone on chance of resumption of cyclicity in acyclic cows [29, 30], associations of the proportion of anoestrous cows by the end of the voluntary waiting period for each herd with the outcomes were not significant in this study. Beneficial influences of progesterone supplementation have been shown elsewhere [26–28], but the metrics presented could not be applied to the parameter values used in our simulation. Any simulation of a biological system must have boundaries, and we restrict our analyses to the farm level, consistent with the major source of greenhouse gas emissions associated with dairy production [37]. In summary, this paper emphasises the importance of being able to predict economic and environmental outcomes in order to facilitate decision making, and justify controversial management practices for society. However we emphasise the importance of simulating specific herd scenarios when making these predictions.

## References

[1] Anon. World Population Prospects: The 2012 Revision: United Nations; 2012 [cited 27 June 2014]. Available: http://esa.un.org/unpd/wpp/index.htm.

[2] Steinfeld H, Gerber P, Wassenaar T, Castel V, Rosales M, de Haan C. Livestock's Long Shadow. Environmental Issues and Options: FAO; 2006 [cited 1 September 2014]. Available: http://www.fao.org/docrep/010/a0701e/a0701e00.HTM.

[3] Foresight. The future of food and farming: Final Project Report: The Government Office for Science; 2011 [cited 27 June 2014]. Available: https://www.gov.uk/government/publications/future-of-food-and-farming.

[4] Wilkinson JM. Re-defining efficiency of feed use by livestock. Animal. 2011; 5: 1014–22. 10.1017/S175173111100005X 22440097

[5] Herrero M, Gerber P, Vellinga T, Garnett T, Leip A, Opio C, et al Livestock and greenhouse gas emissions: The importance of getting the numbers right. Anim Feed Sci Technol. 2011; 166–167: 779–82.

[6] Knapp JR, Laur GL, Vadas PA, Weiss WP, Tricarico JM. Invited review: Enteric methane in dairy cattle production: Quantifying the opportunities and impact of reducing emissions. J Dairy Sci. 2014; 97: 3231–61. 10.3168/jds.2013-7234 24746124

[7] Caraviello DZ, Weigel KA, Fricke PM, Wiltbank MC, Florent MJ, Cook NB, et al Survey of management practices on reproductive performance of dairy cattle on large US commercial farms. J Dairy Sci. 2006; 89: 4723–35. 17106104 10.3168/jds.S0022-0302(06)72522-X

[8] Higgins HM, Ferguson E, Smith RF, Green MJ. Using hormones to manage dairy cow fertility: The clinical and ethical beliefs of veterinary practitioners. PLoS ONE. 2013; 8: e62993 10.1371/journal.pone.0062993 23638174 PMC3637166

[9] Lips M, Relder P. Abolition of raw milk quota in the European Union: A CGE analysis at the member country level. J Agr Econ. 2005; 56: 1–17.

[10] Archer SC, Mc Coy F, Wapenaar W, Green MJ. Bayesian evaluation of budgets for endemic disease control: An example using management changes to reduce milk somatic cell count early in the first lactation of Irish dairy cows. Prev Vet Med. 2014; 113: 80–7. 10.1016/j.prevetmed.2013.10.011 24231116

[11] Hudson CD, Huxley JN, Green MJ. Using simulation to interpret a discrete time survival model in a complex biological system: Fertility and lameness in dairy cows. PLoS ONE. 2014: e103426. 10.1371/journal.pone.0103426 PMC4125137 25101997

[12] Giordano JO, Fricke PM, Wiltbank MC, Cabrera VE. An economic decision-making support system for selection of reproductive management programs on dairy farms. J Dairy Sci. 2011; 94: 6216–32. 10.3168/jds.2011-4376 22118110

[13] De Vries A. Economic value of pregnancy in dairy cattle. J Dairy Sci. 2006; 89: 3876–85. 16960063 10.3168/jds.S0022-0302(06)72430-4

[14] Giordano JO, Kalantari AS, Fricke PM, Wiltbank MC, Cabrera VE. A daily herd Markov-chain model to study the reproductive and economic impact of reproductive programs combining timed artificial insemination and estrus detection. J Dairy Sci. 2012; 95: 5442–60. 10.3168/jds.2011-4972 22916951

[15] Spiegelhalter DJ, Abrams KR, Myles JP. Bayesian approaches to clinical trials and health-care evaluation Chichester, UK: Wiley 2004.

[16] Hudson CD, Bradley AJ, Breen JE, Green MJ. Associations between udder health and reproductive performance in United Kingdom dairy cows. J Dairy Sci. 2012; 95: 3683–97. 10.3168/jds.2011-4629 22720926

[17] R-Development-Core-Team. R: A language and environment for statistical computing: R Foundations for Statistical Computing; 2010 [cited 1 May 2014]. Available: http://www.R-project.org.

[18] Remnant JG, Green MJ, Huxley JN, Hudson CD. Variation in the inter-service intervals of UK dairy cows. J Dairy Sci. 2015; 98: 889–97. 10.3168/jds.2014-8366 25529414

[19] Forde N, Beltman ME, Lonergan P, Diskin M, Roche JF, Crowe MA. Oestrous cycles in Bos taurus cattle. Anim Reprod Sci. 2011; 124: 163–9. 10.1016/j.anireprosci.2010.08.025 20875708

[20] Madouasse A, Browne WJ, Huxley JN, Toni F, Green MJ. A semi-parametric model for lactation curves: Development and application. Prev Vet Med. 2012; 105: 38–48. 10.1016/j.prevetmed.2012.02.009 22391019

[21] AFRC. Energy and protein requirements of ruminants An advisory manual prepared by the AFRC Technical Committe on Responses to Nutrients Wallingford, UK: CABI International; 1993.

[22] Garnsworthy PC. The environmental impact of fertility in dairy cows: A modelling approach to predict methane and ammonia emissions. Anim Feed Sci Technol. 2004; 112: 211–23.

[23] Pursley JR, Mee MO, Wiltbank MC. Synchronization of ovulation in dairy cows using PGF2α and GnRH. Theriogenology. 1995; 44: 915–23. 16727787 10.1016/0093-691x(95)00279-h

[24] Nebel RL, Jobst SM. Evaluation of systematic breeding programs for lactating dairy cows: A Review. J Dairy Sci. 1998; 81: 1169–74. 9594406 10.3168/jds.S0022-0302(98)75679-6

[25] Mawhinney I, Biggadike H, Drew B. Field trial of a planned breeding regimen for dairy cows, using gonadotrophin-releasing hormone and prostaglandin F2α. Vet Rec. 1999; 145: 551–4. 10609573 10.1136/vr.145.19.551

[26] El-Zarkouny SZ, Cartmill JA, Hensley BA, Stevenson JS. Pregnancy in dairy cows after synchronized ovulation regimens with or without presynchronization and progesterone. J Dairy Sci. 2004; 87: 1024–37. 15259238 10.3168/jds.S0022-0302(04)73248-8

[27] McDougall S. Effects of treatment of anestrous dairy cows with gonadotropin-releasing hormone, prostaglandin, and progesterone. J Dairy Sci. 2010; 93: 1944–59. 10.3168/jds.2009-2305 20412908

[28] Stevenson JS, Pursley JR, Garverick HA, Fricke PM, Kesler DJ, Ottobre JS, et al Treatment of cycling and noncycling lactating dairy cows with progesterone during Ovsynch. J Dairy Sci. 2006; 89: 2567–78. 16772576 10.3168/jds.S0022-0302(06)72333-5

[29] Chebel RC, Al-Hassan MJ, Fricke PM, Santos JEP, Lima JR, Martel CA, et al Supplementation of progesterone via controlled internal drug release inserts during ovulation synchronization protocols in lactating dairy cows. J Dairy Sci. 2010; 93: 922–31. 10.3168/jds.2009-2301 20172212

[30] Xu ZZ, Burton LJ. Estrus synchronization of lactating dairy cows with GnRH, progesterone, and prostaglandin F2α. J Dairy Sci. 2000; 83: 471–6. 10750104 10.3168/jds.S0022-0302(00)74905-8

[31] Xu ZZ, Burton LJ, Macmillan KL. Reproductive performance of lactating dairy cows following estrus synchronization regimens with PGF2α and progesterone. Theriogenology. 1997; 47: 687–701. 16728021 10.1016/s0093-691x(97)00027-7

[32] Macmillan KL, Day AM. Prostaglandin F2α —a fertility drug in dairy cattle? Theriogenology. 1982; 18: 245–53. 16725745 10.1016/0093-691x(82)90001-2

[33] Rasbash J, Steele F, Browne WJ, Goldstein H. A User’s Guide to MLwiN, v2.26 Centre for Multilevel Modelling, University of Bristol, UK 2012.

[34] DairyCo. Dairy statistics an insiders guide: DairyCo; 2014 [cited September 16, 2014]. Available: http://www.dairyco.org.uk/resources-library/market-information/dairy-statistics/dairy-statistics-an-insiders-guide-2014/#.VBgZpfldU1I.

[35] Anon. Inventory of U.S. greenhouse gas emissions and sinks: 1990–2011: US Environmental Protection Agency; 2013 [cited 16 September 2014]. Available: http://www.epa.gov/climatechange/Downloads/ghgemissions/US-GHG-Inventory-2013-Main-Text.pdf.

[36] Anon. Measuring effectiveness and cost effectiveness: The QALY: NICE; 2010 [cited 15 December 2014]. Available: https://www.nice.org.uk/proxy/?sourceurl=http://www.nice.org.uk/newsroom/features/measuringeffectivenessandcosteffectivenesstheqaly.jsp.

[37] Foster C, Green K, Bleda M, Dewick P, Evans B, Flynn A, et al Environmental impacts of food production and consumption: A report to the Department for Environment, Food and Rural Affairs Mancheter Business School/Department for Environment, Food and Rural Affairs, London 2006.

